# Supplementary material for: The Phonological Mapping Negativity (PMN) as a language-specific component: Exploring responses to linguistic vs musical mismatch
Source: PLoS One. 2024 Dec 19;19(12):e0315537. doi: 10.1371/journal.pone.0315537 (PMC11658511; doi:10.1371/journal.pone.0315537)

**Topography plots for the individual conditions in time periods as denoted by the clusters (phoneme: 297-401 ms; and note: 259-348 ms)**

Phoneme match:


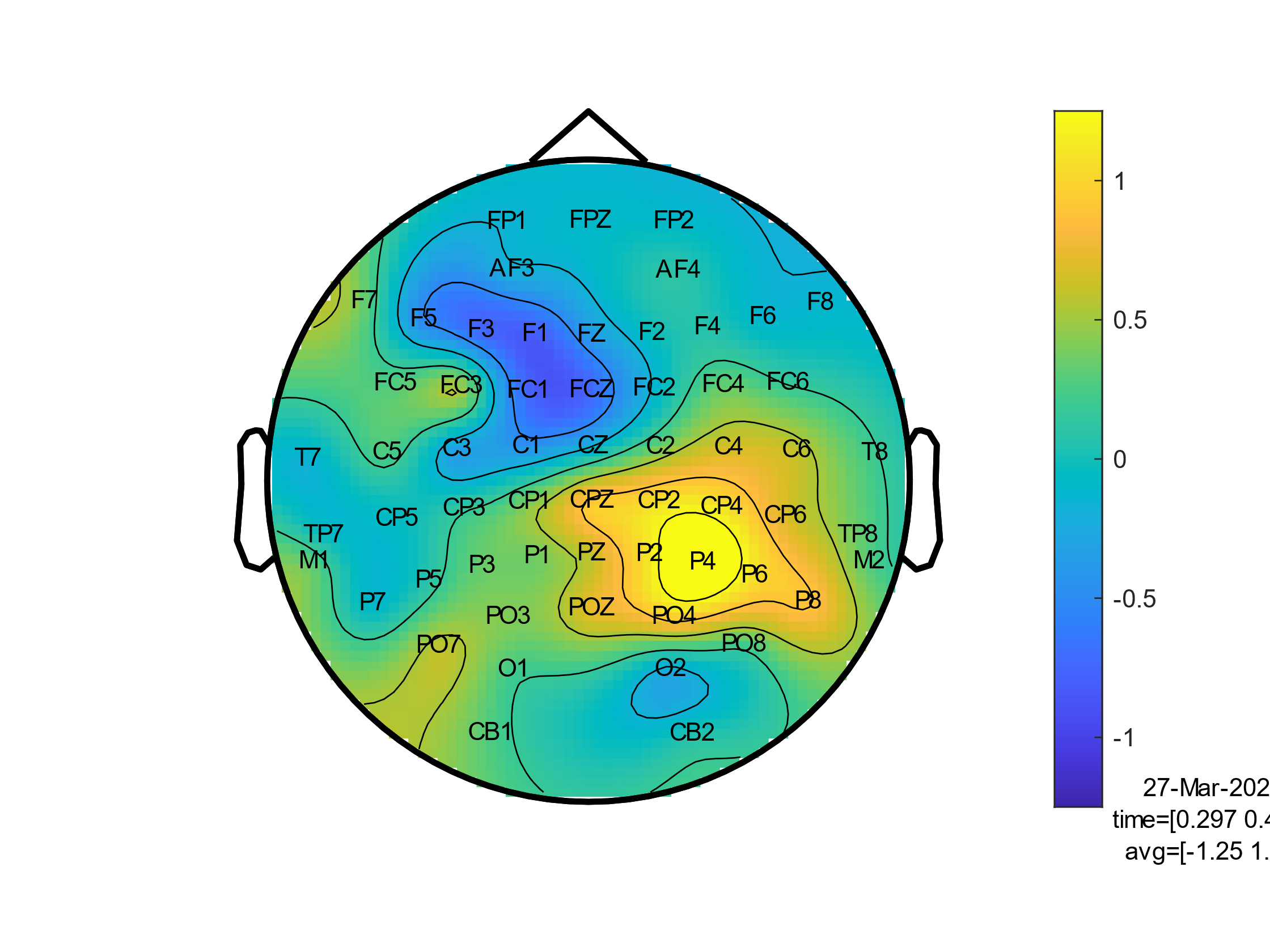


Phoneme mismatch


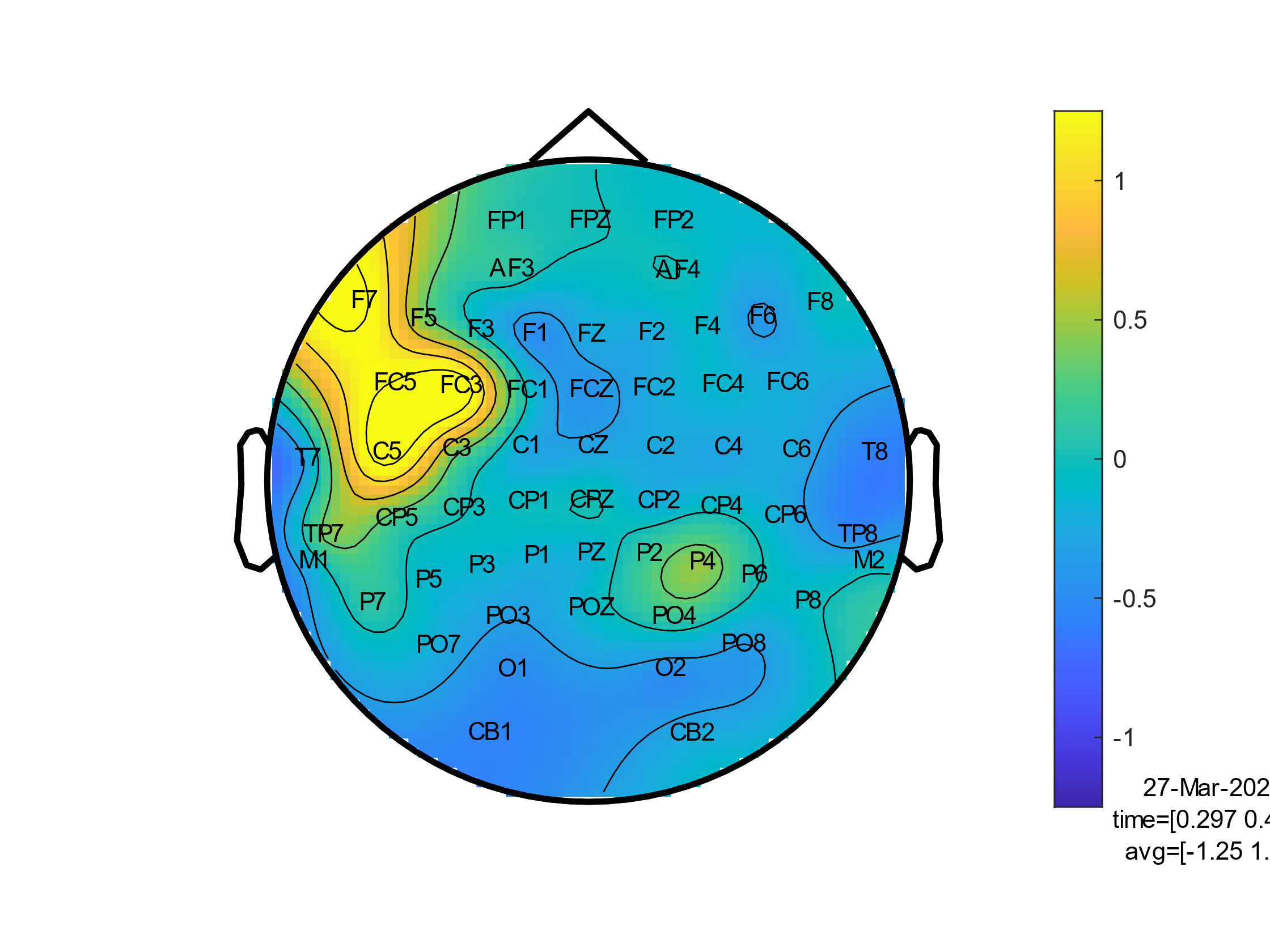


Tone match


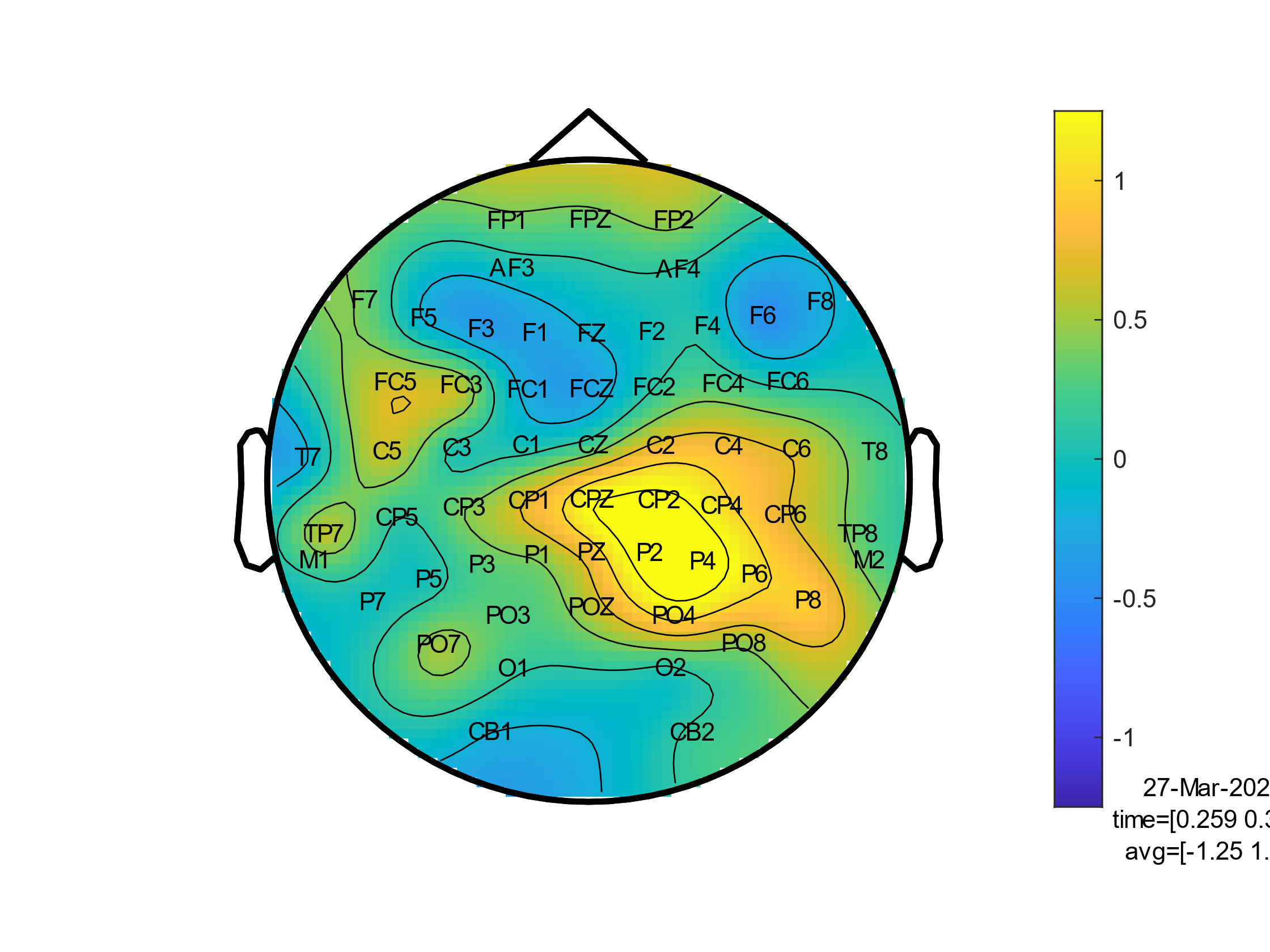


Tone mismatch


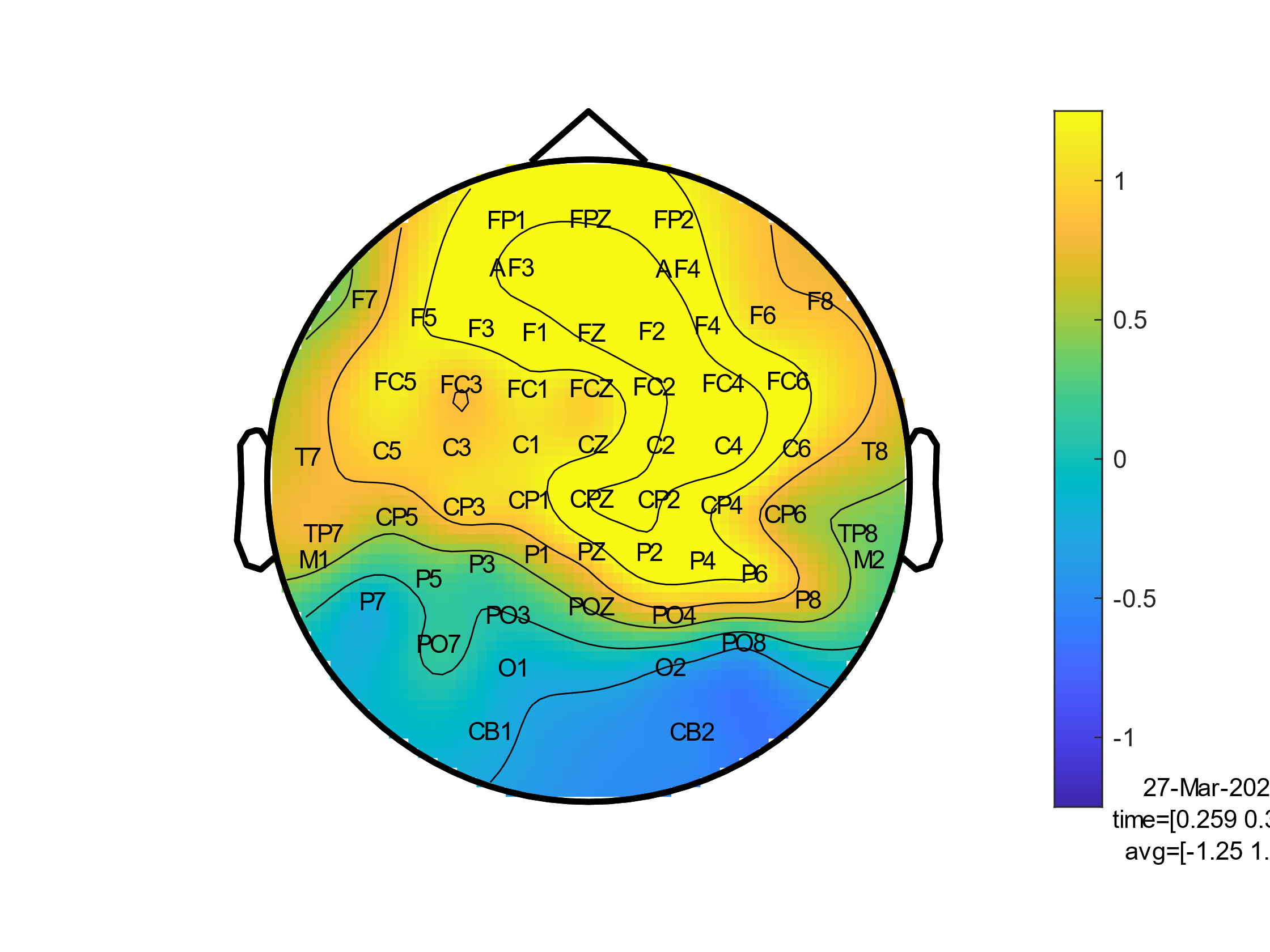

Supplement: S3 File — (DOCX) [file pone.0315537.s003.docx]
