# Supplementary material for: The Phonological Mapping Negativity (PMN) as a language-specific component: Exploring responses to linguistic vs musical mismatch
Source: PLoS One. 2024 Dec 19;19(12):e0315537. doi: 10.1371/journal.pone.0315537 (PMC11658511; doi:10.1371/journal.pone.0315537)
Supplement: Attachment — Submitted filename: Reviewer Response.docx [file pone.0315537.s004.docx]

1. Please ensure that your manuscript meets PLOS ONE's style requirements, including those for file naming. The PLOS ONE style templates can be found at 
[https://journals.plos.org/plosone/s/file?id=wjVg/PLOSOne_formatting_sample_main_body.pdf](https://urldefense.proofpoint.com/v2/url?u=https-3A__journals.plos.org_plosone_s_file-3Fid-3DwjVg_PLOSOne-5Fformatting-5Fsample-5Fmain-5Fbody.pdf&d=DwQGaQ&c=slrrB7dE8n7gBJbeO0g-IQ&r=_2NHSJxa9puNQWOUDnthYw&m=fujAcrHsESopRl5T0bvtzp7KhJbMT1Uy76T430MY_IJNX5uA8RynEewtowHhvTLs&s=SZ_9_77-R9B_Gi94hWdvEt71UIq4IAWk6Tl2sf7lFHQ&e=) and 
[https://journals.plos.org/plosone/s/file?id=ba62/PLOSOne_formatting_sample_title_authors_affiliations.pdf](https://urldefense.proofpoint.com/v2/url?u=https-3A__journals.plos.org_plosone_s_file-3Fid-3Dba62_PLOSOne-5Fformatting-5Fsample-5Ftitle-5Fauthors-5Faffiliations.pdf&d=DwQGaQ&c=slrrB7dE8n7gBJbeO0g-IQ&r=_2NHSJxa9puNQWOUDnthYw&m=fujAcrHsESopRl5T0bvtzp7KhJbMT1Uy76T430MY_IJNX5uA8RynEewtowHhvTLs&s=cxmlgnjXb_sE2W5SXfqwwmd86sJw4Rncw25pAObMYek&e=)

2. Please ensure that you refer to Figure 4 in your text as, if accepted, production will need this reference to link the reader to the figure.

**Response:** apologies for the formatting issues, these should be resolved now.

**Point-by-point response**

5. Review Comments to the Author

Please use the space provided to explain your answers to the questions above. You may also include additional comments for the author, including concerns about dual publication, research ethics, or publication ethics. (Please upload your review as an attachment if it exceeds 20,000 characters)

*Reviewer #1: The article addresses a theoretically well motivated question on the field of the electrophysiological study of linguistic processes: is the Phonological Mismatch Negativity (PMN) ERP component language specific? Moreover, is the PMN distinct from other similar ERP components such as the MMN and N400? The authors claim that the results of the study provides a definitive answer to the first question, but leave the answer to the second question to further studies. I appreciate the importance of the research question investigated in the study, but I have some doubts whether the methodology applied could in fact provide decisive answers to this question.
The article is a concise and well written summary of the study, with some minor typos (see later). The introduction presents a compelling summary of the theoretical background of the PMN component and clearly highlights the theoretical and methodological challenges related to this ERP component. The authors propose a new experimental paradigm to investigate the language specificity of the PMN. This paradigm is markedly different from the paradigms used previously to elicit the PMN. The authors present a lengthy analysis in the introduction about the controversies with the previous paradigms and their reliability to elicit the PMN. I think this is a particularly valuable section of the article. In the following, I present my comments on the article separated to major and minor issues.*

**General response:** We thank the reviewer for the time they have afforded our submission and the constructive comments and suggestions they offer. We particularly appreciate the depth with which the reviewer has considered the methodology of our paper and its overall aims, alongside proposed amendments which we feel have notably improved the manuscript. We have sought to implement the suggested changes where possible, or respond to points in a manner that provides further clarity. We hope that our revisions to each point, laid out below, satisfy the reviewer’s questions and concerns.

*Major issues*
*Experimental paradigm. As I mentioned previously, I have doubts if the paradigm used can provide reliable results related to the linguistic nature of the PMN. My concern is the simultaneous presentation of the linguistic and non-linguistic stimuli. While I think that this a clever way to control for confounds present in previous experiments, I wonder if the ERPs to a complex simultaneous stimulus aggregate are comparable to the ERPs obtained as a correlate of purely linguistic information. I think the authors need to provide a more convincing argument that the results of the present study are comparable to the results of previous studies. Furthermore, even if attention is directed to only one information (either speech of notes), there might be a pre-attentive processing of the non-attended information, which is in fact a way to elicit the MMN. How can the authors make sure that this pre-attentive processing is not manifested in the ERPs somehow?*

**Response:** We acknowledge and appreciate the theoretical grounding of these questions, but we are unsure that the data we present support the points made. The reviewer’s first concern is that ERP responses to simultaneously presented tone and phoneme mismatches might not be comparable to simple phoneme matches. We agree that this is a legitimate question, and would warrant consideration of the validity of our findings had we not observed a reliable PMN response to the phoneme mismatches. Granted, we make a point of observing that the PMN effect we report is notably centro-parietal (as compared to the more typical characterization of the response as fronto-central), but given that there is widespread evidence of centro-parietal PMN effects in literature manipulating simple phoneme match/mismatch (noted in the text), this possibility - that our paradigm might not have succeeded in producing a PMN response that is sufficiently comparable to other literature - doesn’t seem like a criticism that is supported by our findings. From a slightly more theoretical perspective, the point could also perhaps be made that all linguistic stimuli are multidimensional, so a spoken word, for example, is in itself a “complex stimulus aggregate” (consisting of many phonetic features overlapping) just as our stimuli are.

In the latter half of this concern, the reviewer suggests that pre-attentive processing of the non-target stimuli might influence the ‘tone only’ or ‘phoneme only’ conditions. Again, this seems unlikely given the results of a previously published paper (<https://people.ku.edu/~sjpa/pubs/Lewendonetal2023.pdf>) in which we establish that eliciting the PMN in pre-attentive contexts does not appear possible. Furthermore, it should be noted that the present experimental paradigm didn’t have the oddball ratio necessary to elicit MMN responses.

Given the above, and the implications of using a design in which stimuli are not comparable across conditions, it feels to us that although presenting phoneme and tone stimuli concurrently might come with its own limitations, not doing so would result in a notably less well-controlled paradigm in which any effects might be attributed to a host of differences in perceptual qualities of the stimuli.

Minor issues
*1. Introduction: I miss from the introduction a more detailed functional characterisation of the PMN component. The authors mention the PMN “indexes phonological processing at the pre-lexical level”, but this is a very broad description. What does phonological processing mean? What are the input-output processes involved here? I think the article would benefit from a more thorough explanation of the possible functions of PMN in the Introduction, and could help the authors to situate the PMN in the Bornkessel-Schlesewsky and Schlesewsky account in the Discussion.*

**Response:** One of the biggest problems with the PMN is that it is a notably poorly defined and poorly understood component. Here we quote the original characterizations of the PMN (‘phonological processing at the pre-lexical level’) as it was/has been explained in past literature, but go on to highlight the fact that- in reality the effect is badly documented and understood both in terms of its spatiotemporal characteristics and functional significance. It is difficult to elaborate on a characterization of the component produced by others, and that - through the course of the paper - we note the opacity of. However, this comment has been particularly helpful in ensuring we emphasize the lack of consistent characterization of the PMN early on – and we have amended the language of the characterization (P3, lines 57-58; 62-66) to make it clearer that we approach this characterization with caution.

2. *Participants: out of 52 participants, 20 were excluded because of low accuracy in the task. This is a huge number of participants excluded, and raises a questions about the reliability of the paradigm, i.e., if the task was too difficult for a large number of participants. I think the authors should discuss this aspect of the study. Furthermore, some of the participants were left handed. Given that the hemispheric localisation of language related brain areas is unknown in left handed participants, I think it’s not a good idea to include them in the sample.*

**Response:** First, accuracy. We do think that the task was more difficult than anticipated, and this isn’t something we would dispute at all. However, we don’t necessarily feel this implies that the paradigm is not reliable, nor does it render the results redundant. Hypothetically, if we were to assume the paradigm was indeed entirely too difficult to elicit mismatch responses, this should manifest as a complete lack of responses to both phoneme and tone mismatch. Given that reliable responses were found, it seems reasonable to assume although the paradigm was more difficult than we anticipated, participants still detected both types of mismatch. However, based on both this comment we have rerun the analysis including:

1. Only the high accuracy participants and only correct trials (note that the accuracy thresholds differ from those reported in the original paper);

Note mismatch	- positive effect (*p* = .073)

- negative effect (NS)

Phoneme mismatch	- positive effect (NS)

- negative effect (*p* = .049) *

2. both the high and low accuracy participants (correct only trials)

Note mismatch	- positive effect (*p* = .032) *

- negative effect (NS)

Phoneme mismatch	- positive effect (NS)

- negative effect (*p* = .015)*

3. High and low accuracy participants with both correct and incorrect trials included (see full paper).

Note mismatch	- positive effect (*p* = .028) *

- negative effect (NS)

Phoneme mismatch	- positive effect (NS)

- negative effect (*p* = .008) *

Because of the manner in which the effects pan out, we attribute the retention in the trend of effects, but loss of significance across exclusions as an effect of reduced power. Given that the same trends appear across the data, and that exclusion of the high proportion of participants caused concern for both Reviewer 1 and 2, we have instead decided to report results for all participants, irrespective of accuracy, with a footnote to mention that when excluding the low-accuracy participants and/or incorrect trials, and/or left-handed participants, the same general qualitative pattern of results was obtained but didn’t always reach statistical significance.

*3. Figure 1.: While the figure is clear and informative, I wonder if the order of presentation of the target and prime could be changed to better reflect the temporal relations of the two stimuli? I.e., the prime is followed by the target, not the other way around.*

**Response:** We agree. To try and better convey order of presentation we have amended this figure sure that the prime is above the target.

*4. Data pre-processing: The authors applied a low-pass filter of 30 Hz on the segmented data as a final step during the pre-processing. My understanding is that filtering is more reliable when applied on continuous data. Could the authors explain the rationale why not using the low-pass filter together with the high-pass filter?*

**Response:** We have rerun the preprocessing to move the low-pass filter prior to segmentation. All results and figures now reflect this change, and lines 354-355 (P9) have been updated accordingly.

Typos
*p2.para3: “disassociating the PMN from= from the ERP” - ?
p3. para1: “other processing mechanisms Despite considerable “ - missing “.”
p4. para1: “on the PMN amplitude `cannot” - ‘
p6. para1: “featured note which were either correct” - notes
p6. para2: "the presence of a PMN effect for linguistic manipulations in Besson and Macar cannot be conclusively determined” - Besson and Macar study?
p8. para2: “sound-calling earphones” - maybe noise cancelling? why is this important? Can you specify the type of earphones used?
p13. para2: “presented with familiar melodies simultaneously to word” - ?*

**Response:** Thank you!

*Reviewer #2: SUMMARY:
The study investigates the nature of the Phonological Mismatch Negativity (PMN), in particular its language-specificity, and thereby by proxy its relation to the N400 and the MMN components.
This is done using a phoneme-deletion paradigm (which has previously been used for investigating the PMN) in combination with a parallel note-deletion (musical) paradigm.
The study finds support for language-specificity of the PMN via significant negative deflections in the phonological mismatch condition, but not in the musical mismatch condition.


GENERAL COMMENTS:
I wish to congratulate the authors on a great paper. The study is neatly and concisely motivated in the introduction. The paper is clearly and well written throughout. The (full) study itself has an elegant design and the results (that are included) are clearly reported. I also greatly appreciate the supplementary material.
I do, however, have two main concerns that I'd like to see addressed before I can recommend publication.*

**General response:** We first would like to thank the reviewer for the time and consideration they have afforded our paper, and really appreciate their kind words regarding the paradigm and design. The level of attention the reviewer has given to our manuscript is incredibly apparent in the detailed and comprehensive points, corrections and comments, both major and minor. The suggestions provided are insightful and have – wherever possible – been fully implemented to the overall benefit of the paper. Where this has not been feasible, we have tried our best to outline the rationale to the reviewer and make any amendments to the paper that reflect any concerns. At times the comments of Reviewer 2 overlap with those of Reviewer 1, but result in different suggestions in terms of revisions proposed to resolve these issues. Where this has arisen, we have attempted to revise analyses to address the underlying concerns, albeit varying in whether the implementation of suggestions sways more towards the recommendations of one reviewer over the other.

MAIN CONCERN(S):
1. *First off, the comprehensive introduction introduces and motivates the PMN as a component that could potentially be independent of both the MMN and the N400. And the elegant design of the study even supports this endeavour of disentangling the PMN from the two other components, but then only the "speech-specific" aspect of the design is addressed in this study - the attentional aspect is completely ignored (albeit directly integrated in the design). In the Methods section, one can then deduce that the attentional aspect is addressed in a parallel paper. I must admit I struggled quite a bit to come to terms with this decision - and I'm not sure I fully have come to terms with it. There are so many good reasons for integrating the two papers into one (especially since the results from the other paper can't be compared to this one due to radically differences in preprocessing/analysis, i.e. number of participants included). My suggestion is therefore to integrate the attentional contrast in this study as well and just clearly disclaim that those data have already been published (together with another experiment). It would make the integration and interplay of the premise and design of the study stand so much stronger, and thus the conclusions would form a much more coherent picture.*

We acknowledge this point and feel that it is a valid question to ask regarding the presentation of our findings. Our rationale for splitting the results into separate papers was predominantly driven by an attempt not to muddy the waters. The question of whether the PMN is distinguishable from the N400 has received quite some attention over the years. On the other hand, whether the PMN might result from the same underlying mechanism as the MMN is far less represented/tested/questioned (in fact we’re not aware of any other papers apart ours - which forms the inattentive part of this paradigm – that ask this). Instead, the independence of these two components seem to be simply assumed and accepted.

Splitting our results into two separate papers allowed us to fully hone in on the key functional sensitivities of the PMN relied upon to propose its independence from these two other components. In the inattentive PMN paper we question the longstanding assumption that the PMN cannot occur inattentively, a key characteristic that hasn’t before been questioned. In the present paper, we instead hone in on the assumed phonology-specific nature of the PMN that drives PMN/N400/MMN independence claims. Our concern was that attempting to disentangle 3 decades of ‘PMN vs N400’ pm the basis of its status as a phonology-specific response, before – within the same paper- moving into questions of whether the PMN, N400 and MMN are distinct on the basis of attention might lose the reader (in fact, a previous review in which we highlight major issues with prior PMN vs N400 literature has been overwhelmingly cited in *support* of an independent PMN response..).

Instead, we have (as you suggest) made a clear note of the inattentive PMN results of the paradigm, as now published elsewhere. See p15 lines 486-489.

*2. The fact that 20 (out of 52 - or 54? - participants) scored below 50% accuracy seems quite concerning - especially since this number was much lower in the parallel study where only the phonemic mismatches were considered. It tentatively suggests that the tonal mismatch part of the task was harder than the phonemic one (which seems a bit counter-intuitive to me after having listened to the stimuli). Under all circumstances, I strongly urge you to do an by-item analysis on the behavioral results as well to see whether that provides any relevant insight (e.g. if certain match/mismatch items were particularly hard to identify, some of the correct responses for those items in the included participants may not be fully credible)
And then conduct a supplementary analysis with no participants or trials rejected on the basis of incorrect responses to see if and how much that actually matters for the results.
On the note of the by-item analysis, in the Methods it says "The rationale for reducing note duration was to ensure that the processing of phonological input was not impeded by the tones." > But since the onset of the syllables are the only distinguishable cue for the phoneme-mismatches, is it not a bit unfortunate that the tones and syllables onset simultaneously? My impression when listening to the stimuli was this somewhat impedes the processing of the very rapid transition cues in the onsets of the syllables - I could even imagine this being particularly difficult for specific sounds, hence an even greater need for a by-item analysis of the behavioral responses. I don't think, however, that the joint onsets could be the root of the potentially more difficult tonal condition - since the notes are sustained for longer periods of time than the syllable onsets.*

**Response:**

First the simultaneous onset of the phoneme and tones: whilst we agree that this might have reduced the clarify of the syllable onset, we could not think of a better way to produce tone and phoneme mismatches using identical stimuli across conditions. Given the potential for marked waveform differences to result from minor perceptual differences between stimuli, retaining identical stimuli felt like a greater priority that maximizing the ease of mismatch detection (i.e, violating the former posed an increase risk of Type 1 error, whilst the latter increased our chance of Type 2 error). We agree that this is perhaps not ideal, but we felt this was the less risky strategy.

Second, we do think that the task was more difficult than anticipated - this isn’t something we would dispute at all. However, we don’t necessarily feel this implies that the paradigm is not reliable. Hypothetically, if we were to assume the paradigm was indeed entirely too difficult to elicit mismatch responses, this should manifest as a complete lack of (or very weak) response to both phoneme and tone mismatch. Given that reliable responses were found across both types of mismatch, it seems reasonable to assume although the paradigm was more difficult than we anticipated, participants still detected both tone and phoneme mismatch. Based on this comment, and those of review 1, we have rerun the analysis including:

1. Only the high accuracy participants and only correct trials (note that the accuracy thresholds differ from those reported in the original paper);

Note mismatch	- positive effect (*p* = .073)

- negative effect (NS)

Phoneme mismatch	- positive effect (NS)

- negative effect (*p* = .049) *

2. both the high and low accuracy participants (correct only trials)

Note mismatch	- positive effect (*p* = .032) *

- negative effect (NS)

Phoneme mismatch	- positive effect (NS)

- negative effect (*p* = .015)*

3. High and low accuracy participants with both correct and incorrect trials included (see full paper).

Note mismatch	- positive effect (*p* = .028) *

- negative effect (NS)

Phoneme mismatch	- positive effect (NS)

- negative effect (*p* = .008) *

Because of the manner in which the effects pan out, we attribute the retention in the trend of effects, but loss of significance across exclusions as an effect of reduced power. Given that the same trends appear across the data, and that exclusion of the high proportion of participants caused concern for both Reviewer 1 and 2, we have instead decided to report results for all participants, irrespective of accuracy, with a footnote to mention that when excluding the low-accuracy participants and/or incorrect trials, and/or left-handed participants, the same general qualitative pattern of results was obtained but didn’t always reach statistical significance.

*3. "Within each block 10 items formed the present study..." > Plz explain the procedure/block structure much more clearly, incl. using tables and the like. I've tried very hard to parse this passage (while conferring with the procedure description and tables in other paper), and I still can't fully grasp the numbers and division of conditions between the two papers. Here's where I'm at currently:
The LCN paper made use of the following conditions:
[Attention: Phoneme] > [correct note, correct phoneme] and [correct note, incorrect phoneme]
[Attention: Tune] > [correct note, correct phoneme] and [correct note, incorrect phoneme]
(and thus for both attention modes, did not make use of [incorrect note, correct phoneme] and [incorrect note, incorrect phoneme]

This paper made use of the following conditions:
[Attention: Phoneme] > [correct note, correct phoneme], [correct note, INCORRECT PHONEME] and [incorrect note, incorrect phoneme]
[Attention: Tune] > [correct note, correct phoneme], [INCORRECT NOTE, correct phoneme] and [incorrect note, incorrect phoneme]
(and thus for phoneme and tune attention modes, did not make use of either [incorrect note, correct phoneme] or [incorrect note, correct phoneme], respectively

This can be gathered from this section in the Methods:
"The nature of the design meant that for counterbalancing purposes, participants experienced two additional conditions that were not of relevance to the present study (correct note & incorrect phoneme in the blocks in which participants focussed on the tune, and incorrect note & correct phoneme in the blocks in which participants were directed to attend to the word."

However, in a separate part of the Methods:
"Within each block 10 items formed the present study (i.e, the conditions correct note & correct phoneme, correct note & incorrect, incorrect note & correct phoneme, incorrect note & incorrect phoneme), whilst 10 formed a separate study not reported here."

I see how 10 (out of 20) items were used in the LCN paper cuz they ignored half of the conditions (which would be good to also explicate here, i.e. which conditions were used in that study), but I don't see how that leaves only 10 for this study which only ignored 1/4 of the conditions (namely the [correct note & incorrect phoneme] in the Phoneme-attention mode and the [incorrect note & correct phoneme] in the Tune-attention mode)?
Furthermore, the fact that all four conditions are mentioned in the i.e.-parenthesis makes it rather hard to remember/discern that for the [correct note & incorrect phoneme] and [incorrect note & correct phoneme] this only pertains to the "relevant" attention mode.

In addressing my concerns in point 1 (and partly point 2), the reporting of the design may change slightly, but it would still require a much more clearer explication and visual presentation to be accessible to the readers.

I will re-assess the Discussion once the above points have been addressed.*

**Response:** We have included an additional Table (P9 - Table 2) and hope that this makes the design clear. Perhaps the lack of clarify comes from the exclusion of a match condition (e.g., phoneme match/tone match) where the *other* stimuli feature is a mismatch (i.e., only conditions in which the tone also matched were included as the phoneme match condition). Please do let us know if the table is sufficient or whether the design is still a little confusing and further clarification is required.

*MINOR:

FIGURES:

Figure 2 & 3 captions: topographies are mentioned but not included - I'm assuming they have just been moved to the bottom of Figure 4 and then the captions haven't been updated (or the other way around)?*

**Response:** Thank you, we’ve addressed this now.

*No line or page numbers provided, hence I can only refer to what main sections of the text a given change relates to:*

INTRODUCTION:

*"PMN from= from the" > "PMN from the"*

*"(Van den brink & Hagoort, 2004; Van den Brink et al., 2001; Hagoort & Brown, 2000); Connolly & Phillips, 1994)" > (Van den brink & Hagoort, 2004; Van den Brink et al., 2001; Hagoort & Brown, 2000; Connolly & Phillips, 1994) [i.e. delete the extra closing parenthesis]

"phonologically incongruent" (or "phonological incongruence") > I'm thinking "unexpected" or something similar is better suited here - to me, phonologically incongruent would rather be something like "*shand" in stead of "hand"*

*"In their 2020 review, Lewendon et al." > Since the first author overlaps with the current study, it sounds peculiar to say "their" here, consider rephrasing to acknowledge the link to the current author list more clearly

"PMN amplitude `cannot necessarily be deemed meaningful" > "PMN amplitude cannot necessarily be deemed meaningful" [delete the accent aigu]

""Lewendon, Britton & Politzer-Ahles (under review/forthcoming)" > has now been published and thus needs updating*

*"this shared functional sensitivity, alongside overlapping topography the timing invite questions" > I can't parse this properly - there's probably a comma missing after "topography", but I still need another word or two somewhere before or after "the timing" to make proper sense of the sentence*

*"featured note which were either correct" > "featured notes which ..."*

*"ERP component is* *selectively sensitivity to linguistic manipulations" > "selectively sensitive ..."*

*"the mechanisms that given rise to" > "have given" or "gave"*

*"In contrast, the MMN has been reliably demonstrated ..." > plz consider also including some of the papers on musical multi feature paradigms, e.g.*
[*https://pubmed.ncbi.nlm.nih.gov/21621766/*](https://urldefense.proofpoint.com/v2/url?u=https-3A__pubmed.ncbi.nlm.nih.gov_21621766_&d=DwQGaQ&c=slrrB7dE8n7gBJbeO0g-IQ&r=_2NHSJxa9puNQWOUDnthYw&m=fujAcrHsESopRl5T0bvtzp7KhJbMT1Uy76T430MY_IJNX5uA8RynEewtowHhvTLs&s=m16OgH_aC1Blv2W6Uh9cpIN6FY5AV9rV3kjfvf0Q1TY&e=)
[*https://www.ncbi.nlm.nih.gov/pmc/articles/PMC6990974/*](https://urldefense.proofpoint.com/v2/url?u=https-3A__www.ncbi.nlm.nih.gov_pmc_articles_PMC6990974_&d=DwQGaQ&c=slrrB7dE8n7gBJbeO0g-IQ&r=_2NHSJxa9puNQWOUDnthYw&m=fujAcrHsESopRl5T0bvtzp7KhJbMT1Uy76T430MY_IJNX5uA8RynEewtowHhvTLs&s=r6AHR4twRLEMyPW8Np0RvAvfE8wj88oMEUAvyu3EukY&e=)
 *"word (e.g., Prime: /pɪˈrɑːnə/)" > none of the other examples are accompanied by their labels (e.g. Target), so I suggest opting for consistency in that regard, and then perhaps also spell out the word - not all readers of PLoS ONE can be expected to be fully fluent in parsing phonemic transscriptions.*

*"Directing participant attention to either the notes or linguistic stimuli, we therefore independently manipulated either tone or phoneme match/mismatch, whilst retaining physically identical target stimuli across conditions." > since there aren't any objective measures of directed attention in this setup AND because the performance was so relatively poor in many participants, I would suggest to rephrase this sentence in a less categorical manner - I find it a bit of a push to say that tone or phoneme match/mismatch were "independently manipulated" due to the direction of participant attention.*

**Response:** Done! Thank you for the thorough check of the manuscript in addition to the methodological suggestions.

METHODS:

*"Fifty-two native English speakers took part in the experiment." > In the other paper on the same dataset, 54 initial participants are mentioned - in this one, only 52. Why the discrepancy?*

**Response:** apologies, this is a typo. The correct number is 54. This has been updated.

*"(iv) incorrect note & incorrect phoneme (e.g., ambitious – bitious; A4-B4-C5 – E5-C5)" > "licious"*

*"were directed to attend to the word" > "were directed to attend to the word)" [i.e. close the parenthesis]*

*"Note stimuli were in Sibelius®" > "were recorded/generated in Sibelius®"?*

*"To The mean intensity of each" > "The mean intensity of each"*

*"(i.e, the conditions correct note & correct phoneme, correct note & incorrect," > "correct note & incorrect phoneme,"*

*"data was low-pass filtered 30 Hz" > "filtered at 30 Hz"*

*"A total of 78 trials were removed during artifact rejection" > could the order of the artefact rejection plz be switched. That way the numbers for the incorrect responses reflect how many trials were rejected for the different conditions on that particular basis (to better reflect potential differences between conditions using that criterion), and then the extreme value-based rejection routine can be applied subsequently and a summary of how many trials were left for each condition for statistical analyses can be given.*

*"leaving an average of 38.69/40 trials" > plz provide SD across participants for these averages*

**Response:** Again, thank you.

RESULTS:

*"Overall accuracy was 94% in the phoneme match condition, 99% in the tone match condition, 77% in the tone mismatch condition, and 81% in the phoneme mismatch condition." > I'm assuming that these numbers don't align with the numbers mentioned in the artefact routine because the extreme-value based rejection was run first, right?*

**Response:** Yes

*"The increase in negativity was driven by a cluster occurring approximately 387 - 500 ms across central, parietal and occipital electrodes" > plz make explicit that the proposition "was driven" is based on visual inspection (I presume), and then make a note of the inherent limitations of spatial and temporal conclusions made based on cluster-based permutation tests with a reference to the Sassenhagen & Drasckow paper (*[*https://onlinelibrary.wiley.com/doi/10.1111/psyp.13335*](https://urldefense.proofpoint.com/v2/url?u=https-3A__onlinelibrary.wiley.com_doi_10.1111_psyp.13335&d=DwQGaQ&c=slrrB7dE8n7gBJbeO0g-IQ&r=_2NHSJxa9puNQWOUDnthYw&m=fujAcrHsESopRl5T0bvtzp7KhJbMT1Uy76T430MY_IJNX5uA8RynEewtowHhvTLs&s=fUQhWi8D0HINZfh2Sv0mKvTQOe8PQQirSxcH1nI11hM&e=)*) and perhaps the fieldtrip-note as well (*[*https://www.fieldtriptoolbox.org/faq/how_not_to_interpret_results_from_a_cluster-based_permutation_test/*](https://urldefense.proofpoint.com/v2/url?u=https-3A__www.fieldtriptoolbox.org_faq_how-5Fnot-5Fto-5Finterpret-5Fresults-5Ffrom-5Fa-5Fcluster-2Dbased-5Fpermutation-5Ftest_&d=DwQGaQ&c=slrrB7dE8n7gBJbeO0g-IQ&r=_2NHSJxa9puNQWOUDnthYw&m=fujAcrHsESopRl5T0bvtzp7KhJbMT1Uy76T430MY_IJNX5uA8RynEewtowHhvTLs&s=hTJrt4yikmfSeJvdoRW5V3nxeNsRSN28cJdoDQHwsQ8&e=)*). This cautious wording should then also be applied to the remainder of the temporal and spatial interpretations made in the Results section - incl. the caption to Figure 4 "electrodes labels in black denote electrodes that formed part of the cluster that drives the significant difference between conditions".*

*"across central, parietal and occipital electrodes (Figure 1A)" > "(Figure 4A)"*

*"more positive response than tone match (p = 0.019) (Figure 1B)" > "(Figure 4B)"*


*SUPPLEMENTARY MATERIAL:
Again, huge kudos for providing this material. Really a great help in properly understanding the study.
In the audio files, there are 20 primes (incl. targets) that are not listed in stimulus spreadsheet (and which weren't paired with any incorrect note versions) - what was the role of these stimuli?
atlantic
competing
complaining
completion
computer
condensing
detective
director
enquiry
eviction
exceeding
exciting
extinction
fixation
impressing
inspection
pacific
quotation
umbrella
withdrawal*

**Response:** These are the ten practice trials for the tune and phoneme blocks (2*20)

PRACTICAL COMMENTS:
*Plz provide line (and page) numbers in the revised version of the manuscript*

6. PLOS authors have the option to publish the peer review history of their article ([what does this mean?](https://urldefense.proofpoint.com/v2/url?u=https-3A__journals.plos.org_plosone_s_editorial-2Dand-2Dpeer-2Dreview-2Dprocess-23loc-2Dpeer-2Dreview-2Dhistory&d=DwMGaQ&c=slrrB7dE8n7gBJbeO0g-IQ&r=_2NHSJxa9puNQWOUDnthYw&m=fujAcrHsESopRl5T0bvtzp7KhJbMT1Uy76T430MY_IJNX5uA8RynEewtowHhvTLs&s=mjhx1to9S9Battr34xaI27gW11oSSAIAeorcmGZAfmE&e=)). If published, this will include your full peer review and any attached files.


If you choose “no”, your identity will remain anonymous but your review may still be made public.


**Do you want your identity to be public for this peer review?** For information about this choice, including consent withdrawal, please see our [Privacy Policy](https://urldefense.proofpoint.com/v2/url?u=https-3A__www.plos.org_privacy-2Dpolicy&d=DwMGaQ&c=slrrB7dE8n7gBJbeO0g-IQ&r=_2NHSJxa9puNQWOUDnthYw&m=fujAcrHsESopRl5T0bvtzp7KhJbMT1Uy76T430MY_IJNX5uA8RynEewtowHhvTLs&s=QmspelW7KgwT18xKul_HJL3A-eB4dAK46ANT214Dk74&e=).

Reviewer #1: No

Reviewer #2: **Yes:** Andreas Højlund

[NOTE: If reviewer comments were submitted as an attachment file, they will be attached to this email and accessible via the submission site. Please log into your account, locate the manuscript record, and check for the action link "View Attachments". If this link does not appear, there are no attachment files.]

While revising your submission, please upload your figure files to the Preflight Analysis and Conversion Engine (PACE) digital diagnostic tool, [https://pacev2.apexcovantage.com/](https://urldefense.proofpoint.com/v2/url?u=https-3A__pacev2.apexcovantage.com_&d=DwMGaQ&c=slrrB7dE8n7gBJbeO0g-IQ&r=_2NHSJxa9puNQWOUDnthYw&m=fujAcrHsESopRl5T0bvtzp7KhJbMT1Uy76T430MY_IJNX5uA8RynEewtowHhvTLs&s=6SxRoPDGoZU8f3huaTm61BpSP9eosSyrI_xs9BL_dtg&e=). PACE helps ensure that figures meet PLOS requirements. To use PACE, you must first register as a user. Registration is free. Then, login and navigate to the UPLOAD tab, where you will find detailed instructions on how to use the tool. If you encounter any issues or have any questions when using PACE, please email PLOS at [figures@plos.org](mailto:figures@plos.org). Please note that Supporting Information files do not need this step.
