# Supplementary material for: The Phonological Mapping Negativity (PMN) as a language-specific component: Exploring responses to linguistic vs musical mismatch
Source: PLoS One. 2024 Dec 19;19(12):e0315537. doi: 10.1371/journal.pone.0315537 (PMC11658511; doi:10.1371/journal.pone.0315537)
Supplement: Attachment — Submitted filename: Reviewer Response_round_2.docx [file pone.0315537.s005.docx]

**We would like to thank the reviewer once again for the time that he has taken suggesting improvements to our manuscript. A review of this depth, with its level of constructive and consideration is a no quick endeavor, and we are genuinely grateful to the reviewer for the fact that our paper has benefited from this process not just once but twice. We feel that all the changes he has suggested across the two revisions have strengthened the paper substantially, and thank him for the effort and energy he has expended meticulously going through our manuscript.**

Reviewer #2: GENERAL COMMENTS:
This time around I wish to congratulate the authors on a tremendous effort in addressing all the concerns raised by the other reviewer and myself. Data have been re-analyzed (by also incl. the low-accuracy participants - and the preprocessing has also been changed according to my recommendation).
The result is a much improved paper. Clarity wrt design and stimuli has also improved greatly.

I only have a few minor concerns that I recommend be addressed before I can recommend publication.


MINOR CONCERNS:

PROOFREADING:
During the revision process quite a few typos and the like have crept in, so it seems an extra proofreading is warranted, e.g.:
396: "in negativity most pronounced" > "in negativity [was] most pronounced"
Footnote 3: "didn't" > "did not"
499: "gestures – [65]" > "gestures [65]"

**Thank you for noting these, we’ve made the corrections.**

TABLE 2:
Great addition, thank you.
Regarding readability of the table, I would suggest including a space between P± and N± (and even better, fully aligning all P± and all N±, resp - e.g. via split cells or however, the authors might prefer to do this). For the Y and N column (aka. "Incl. in present study"), I would suggest removing all N and only include Y (as they are listed now, Y and N are quite difficult to visually distinguish, hence either leaving all N blanks, or denote with a hyphen (-), would greatly improve readability).

**As recommended, we have split the condition column into two cells to improve the readability. We would ideally prefer to retain the N in the ‘incl. in present study’ column, but absolutely agree in terms of the readability. To address this, we have changed the font colour of the Ns to grey.**

FIGURES 2-3:
I acknowledge that the authors have chosen to mainly delete references to the temporal extent of the main cluster identified in the statistical analyses in the main text in order to address the concerns I raised with reporting the temporal (and spatial) extent of clusters from cluster-based permutation testing; however, I find that the reader is left at a disadvantage in interpreting the myriad of waveforms (12 per figure) when there's no temporal guidance. Hence, the vertical bars from the original plots could perhaps be re-introduced with a reference to a similar formulation as in Fig 4 (as well as to the raster plots and topographies in Fig 4). And finally I again urge the authors to just include a one-liner mentioning caution in interpreting cluster extent too strongly/directly in cluster-based permutation testing (with a reference to Sassenhagen & Drasckow (2019)).

**Thank you for this, we agree that the amendment to the figure combined with the reference (I’ve placed this directly underneath the cluster figures) will definitely make things clearer for the reader.**

DISCUSSION:
As stated in my first review, I can now assess the Discussion more clearly, given the final results of the revision.
I generally applaud the critical reading of the literature in the Discussion in combination with the contextualization of the results of the present study.
However, I do find that the first section of the Discussion on the topographies (ll 436-445) leaves the reader a little short-handed.
First off, it's hard to see how "Both effects clearly show a frontal positive and posterior negative pattern" - to me, the PMN pattern is much more lateralized than frontal-posterior, so it would be great to explicate this proposed similarity in a way that more clearly guides the reader towards what the authors have in mind.
Next, without any supporting references, the generalization that "Whilst such an inversion of effects – with components consisting of opposite polarities on one side of the head compared to the other is typical of all ERP responses, the similar dipoles but slightly different orientations of the topography plots perhaps warrant further investigation" seems a bit hasty... this is certainly true of MEG data (from magnetometers), but less so of EEG data, esp. if we assume that (at least part of) the observed responses originate in the auditory cortices (then the opposite polarity will often not be detectable cuz we cannot record EEG from the below the brain).
Further, the authors themselves refer later in the Discussion to work on musical mismatches (ll 490-492) that elicit frontal P300-like negativities. This seems very relevant to bring into play here - in order to better understand the frontal positivity elicited in the note condition. Is this most sensibly interpreted as an early P300/P3a?
Finally, how common is this right-posterior negativity of the PMN compared to the literature. How much of this can be related to the concurrent presentation of the musical stimuli? Any literature on how this concurrent presentation affects the topography of these early responses?
Hence, I urge the authors to substantiate the discussion of the differences in topographies considerably (especially considering the above-mentioned references later in the discussion). And then I urge the authors to anchor the speculation regarding "similar dipoles but slightly different orientations of the topography" in some literature in some way - either generally wrt the plausibility of the similarity of the underlying dipoles given the topographies or specifically wrt previous literature on almost identical paradigms.
In all of the above, it's important to bear in mind that the interpreted topographies reflect difference waveforms - and thus, any dipole inferences hinges on the topographies reflecting an underlying "difference/mismatch" response and not just differences in different co-occurring responses. Hence, to guide the reader in all of the above, it would be of great help if the topographies of the underlying responses to the match and mismatch conditions for the reported time window together with the rather clear P1-N1-P2 complex could be visualized either in the main paper or in the supplementary material for reference?


REFERENCES:
Sassenhagen J, Draschkow D (2019). Cluster‐based permutation tests of MEG/EEG data do not establish significance of effect latency or location. Psychophysiology. e13335. [https://doi.org/10.1111/psyp.13335](https://urldefense.proofpoint.com/v2/url?u=https-3A__doi.org_10.1111_psyp.13335&d=DwQGaQ&c=slrrB7dE8n7gBJbeO0g-IQ&r=_2NHSJxa9puNQWOUDnthYw&m=Tc3hCTqM3yU00kmH6FLdk6r4omVdoptckMhfeGlQPmSVltgp5QdcOlL9MbxVyOc7&s=8wX99xGczxWNdA0DyoI9jk1_yLNww9jabQoFk0l6ZlY&e=)

**Rereading this section of the discussion, we agree wholeheartedly with the reviewer that it lacks a degree of detail both in terms of our interpretation and the descriptions of the effects. On reflection, we wondered if the best fit for this paragraph might be later in the discussion, specifically pairing it with the discussion of N400/P300 elicitation in instances of musical mismatch (497-507) and the concluding remarks. We’ve also substantially tamed down a few claims, and tried to reframe this so that it is a little more in-keeping with the general conclusions. Unfortunately it is difficult to cite similar research on the PMN using concurrently presented stimuli or non-linguistic input, because to our knowledge this doesn’t really exist. Beyond this, substantial differences in reporting of timing and topography of the PMN across papers and paradigms (sometimes due to paradigm/manipulation, other times due to recording or preprocessing, and finally, on other occasions not clearly caused by anything at all) mean that we don’t feel particularly confident drawing strong conclusions about the right lateralization of the topography of the effect. Finally, we have included the topography maps for all conditions (not just difference waves) in the supplementary material.**
